# A Reference-Dependent Computational Model of Anorexia Nervosa

**DOI:** 10.3758/s13415-021-00886-w

**Published:** 2021-03-09

**Authors:** Francesco Rigoli, Cristina Martinelli

**Affiliations:** 1 Department of Psychology, City, University of London, Northampton Square, London, EC1V 0HB UK; 2 Kingston University, Penrhyn Road, Kingston Upon Thames, Surrey KT1 2EE UK

**Keywords:** Anorexia, Evaluation, Reference dependent, Perfectionism, Dichotomous thinking, Control

## Abstract

Influential accounts interpret anorexia as arising from perfectionism, dichotomous thinking, and poor control expressed in a variety of life domains, resulting in low self-esteem. In this context, restraining eating would allow patients to re-establish some control and self-esteem. Although this view has offered important insight, one shortcoming is that constructs such as perfectionism, control, and dichotomous thinking, remain poorly specified. To clarify these constructs, we propose a computational model of anorexia. This relies on previous theories of evaluation, which highlight its reference-dependent nature: when attributing a value to an outcome, our brain automatically assesses the outcome relative to its context. Following these theories, the model proposes that a high reference point explains general characteristics such as perfectionism, dichotomous thinking, low self-esteem, and low sense of control. These characteristics would result specifically in anorexia when the sense of control regarding body shape, compared with other life domains, is relatively high. The model raises the possibility that reference effects also might explain why patients pursue extremely low weight; exposure to skinny body images—one product of obsessive dieting—might change the reference point for their own body, hence leading to extremely low body weight, staunch refusal to gain weight, and body misperceptions. The model contributes to clarify key concepts adopted in the literature and their relation. Such computational formulation might help to foster theoretical debate, formulating novel empirical predictions, and integrate psychological and neuroscientific perspectives on anorexia.

## Introduction

Anorexia nervosa (AN) is a form of eating disorder characterised by an obsession with body shape, combined with extremely low weight, staunch refusal to eat, and body misperceptions (American Psychiatric Association, 2013). In conjunction with these symptoms, AN patients manifest characteristics common to other disorders, including perfectionism, dichotomous thinking, low self-esteem, and scarce sense of control (Kaye, Wierenga, Bailer, Simmons, & Bischoff-Grethe, 2013). Influential accounts (Cooper, 2005; Fairburn, Shafran, & Cooper, 1999; Slade, 1982) interpret AN as arising from perfectionism, dichotomous thinking, and poor control expressed in a variety of life domains, resulting in low self-esteem. In this context, controlling body shape by restraining eating would provide patients with the only way to re-establish some degree of control and self-esteem, hence becoming the patients’ primary goal. This view has offered important insight and has been supported empirically. However, one shortcoming is that fundamental constructs such as perfectionism, control, and dichotomous thinking remain somewhat poorly specified. In other words, what do these concepts precisely mean? To address this, factor analysis can be adopted to identify different dimensions underlying these constructs. This data-driven approach is well-established and has contributed substantially to the literature (Bardone-Cone et al., 2007; Byrne, Allen, Dove, Watt, & Nathan, 2008) (e.g., highlighting different forms of control, with only some affected in AN; Froreich, Vartanian, Grisham, & Touyz, 2016). We advocate an alternative, theory-driven, approach, consisting in describing the mechanisms underlying AN adopting computational modelling (Frank et al., 2016). This perspective offers a formal description of the mechanisms involved, potentially providing a clearer definition of concepts classically used in the literature and of their relationship.

At the core of our proposal is the notion of evaluation (the process through which positive or negative value is attributed to the different outcomes), which underlies concepts such as eating behaviour, perfectionism, control, and self-esteem. Evaluation drives “hot” aspects of cognition such as emotion, motivation, affect, and decision-making. Contemporary models of evaluation highlight its reference-dependent nature (Kőszegi & Rabin, 2006; Louie, Glimcher, & Webb, 2015; Louie, Khaw, & Glimcher, 2013; Rigoli, 2019; Rigoli et al., 2016; Stewart, 2009; Stewart, Chater, & Brown, 2006; Woodford, 2012): when attributing a value to an outcome, our brain automatically assesses the outcome not in isolation, but relative to its context. As an example, consider an individual who is purchasing a house and who discovers that the price of the house is £10 more than expected. Compare this with someone paying for a coffee and realising that the price is £10 more than expected. Although objectively both individuals experience an equivalent unforeseen extra-cost of £10, we would expect the second person to be way more upset than the first. This example stresses the idea that evaluation is reference-dependent, namely that the subjective value of outcomes strongly depends on the context where these outcomes are experienced. Our theory builds on this notion, and hence it is referred to as Reference Dependent Model of Anorexia (RDMA). We will see how this framework can shed light on key constructs underlying AN. The next section introduces the computational model. This is followed by a description of how general characteristics (such as perfectionism and scarce control) first, and specific symptoms next, arise. Finally, the model is discussed in relation with previous models of AN and regarding other broad issues.

## The model

Contemporary models of reference-dependency disagree on important issues, but they all agree on fundamental principles (Kőszegi & Rabin, 2006; Louie et al., 2013, 2015; Rigoli, 2019; Rigoli, Friston, et al., 2016; Stewart, 2009; Stewart et al., 2006). Here we will rely on a specific model (Rigoli, 2019, 2021; Woodford, 2012); however, similar arguments would arise if different models were adopted. The reason for focusing on this specific model is that, at least in some domains, this represents one of the major candidates for explaining evaluation (Rigoli, 2019). Moreover, the model is simple and can be easily applied to AN (see below).

Consider an environment or context *k* (e.g., school) where a set of outcomes (e.g., school marks) can be experienced, each associated with a raw value (e.g., the actual mark). For each outcome, the calculation of the subjective value *V*_*R*, *k*_ associated with the raw value *R*_*k*_ depends on the following logistic function:

1 $$ {V}_{R,k}=\frac{1}{1+{e}^{-\kern0.5em \frac{R_k-{\mu}_k}{\sigma_k}}} $$

**Fig. 1:**
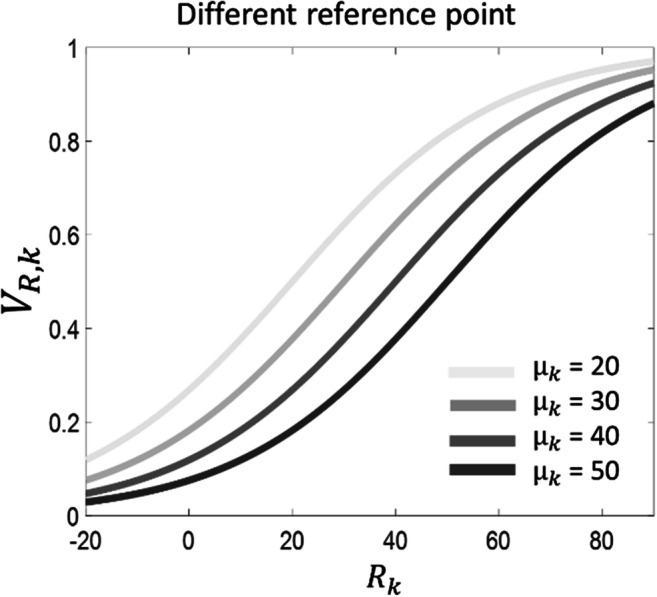
Subjective value as a function of raw value for different reference point *μ*_*k*_ (*σ*_*k*_ = 20 for all lines)

A logistic function (prescribing that the subjective value of a stimulus is 0 < *V*_*R*, *k*_ < 1) has emerged as more appropriate than alternative possibilities (e.g., a linear function; Rigoli, Friston, et al., 2016; Rigoli, 2019) to explain empirical evidence on decision-making (e.g., it can account for context-effects in the curvature of the value function (Rigoli, 2019; Stewart, 2009; Stewart et al., 2006)). The parameters *μ*_*k*_ and *σ*_*k*_ (being *σ*_*k*_ > 0) are the reference point and the uncertainty associated with context *k*, respectively (each context has its own parameters). These parameters capture the reference-dependent nature of evaluation: the subjective value, which is experienced at a subjective level and drives behaviour, is not equivalent to the raw value, but it depends on some reference information. The RDMA proposes that subjective value can be experienced as either reward or punishment, occurring when *V*_*R*, *k*_ > 0.5 and *V*_*R*, *k*_ < 0.5, respectively (a neutral experience occurs when *V*_*R*, *k*_ = 0.5). Based on this definition, note that reward is experienced when *R*_*k*_ > *μ*_*k*_ and punishment is experienced when *R*_*k*_ < *μ*_*k*_. Therefore, the reference point can be interpreted as the standard (or an expectation) associated with a context *k*, to which outcomes are compered to and are evaluated as reward (i.e., better than the standard) or as punishment (i.e., worse than the standard) (Fig. 1). For example, the reference point *μ*_*k*_ might indicate the standard mark at school, implying that a better mark will be perceived as success and a worse mark as failure. The parameter *σ*_*k*_ can be interpreted as the level of uncertainty about the own standard, prescribing how much a discrepancy from the reference point will be weighted. In other words, it determines how subjectively good or bad an outcome is compared with the reference point. For example, if one has received a mark above/below the standard, the uncertainty parameter determines how subjectively good/bad the mark is. If there is high uncertainty, then a discrepancy will not be weighted much, minimizing the subjective distance from the reference point. Hence, the mark above/below the standard will not be considered too good/bad. Conversely, if there is low uncertainty, a discrepancy will be weighted heavily, maximising the subjective distance from the reference point. Hence, the mark above/below the standard will be considered as very good/bad.

Based on equation 1, an individual can evaluate a variety of states within a context, such as the current, past, and future state of affair. Three of such evaluations are particularly relevant: 1) *V*_*pres*, *k*_, capturing the subjective value attributed to the current state of affair (e.g., the current performance at school); 2) *V*_*act*, *k*_, capturing the subjective value attributed to the future outcome achievable by performing appropriate actions (e.g., the performance at school achievable with proper commitment); 3) *V*_*Noact*, *k*_, capturing the subjective value attributed to the future outcome expected without performing those appropriate actions (the performance at school expected without much commitment) (note that, by definition, *V*_*act*, *k*_ > *V*_*Noact*, *k*_). The RDMA proposes that these three evaluations are at the root of both self-esteem and control. Self-esteem reflects the level of satisfaction about the current general state of the self (Branden & Archibald, 1982). Based on this, the RDMA defines self-esteem as equal to the subjective value associated with the current state (*V*_*pres*, *k*_) averaged across all contexts. Control indicates to what degree one expects to achieve goals with appropriate actions (Dayan, 2012; Maier & Seligman, 1976; Rigoli, Pezzulo, & Dolan, 2016; Seligman, 1974). Following this definition, control for context *k* can be defined as:

2 $$ {C}_k={V}_{act,k}-{V}_{Noact,k} $$

This corresponds to the subjective value expected by performing appropriate actions minus the value expected without those actions. While *C*_*k*_ describes the control associated with a specific context *k*, a general control can be derived by averaging control across all contexts (Rigoli, Pezzulo, & Dolan, 2016). The RDMA proposes that control is critical when deciding which context one should engage with: contexts associated with higher control would be more likely to attract engagement. For example, if one perceives higher control in the context of sport compared with the context of school, the person will engage in sport and disregard school. Intuitively, this captures the idea that people are attracted by contexts where they believe that their condition can be improved.

In short, thanks to the reference point *μ*_*k*_ and the uncertainty parameter *σ*_*k*_, the RDMA highlights the reference-dependent nature of subjective value. From this model, a formal definition of self-esteem and control can be proposed. Below, we will explore how this model of evaluation can be applied to explain AN.

## General characteristics

Consider an example of a context *k* where an agent can experience four possible raw values (10, 30, 50, 70), and where the contextual average and SD are 40 and 10, respectively. The RDMA suggests that, within this context, adaptive evaluation occurs if the reference point *μ*_*k*_ corresponds to the contextual average (equal to 40 in this example) and the uncertainty parameter *σ*_*k*_ corresponds to the contextual standard deviation SD (equal to 10 in this example) (Rigoli, 2019). In other words, adaptive evaluation occurs when an individual has a realistic representation of the context and its statistics and uses this representation to evaluate each stimulus appropriately relative to the others. Conversely, when the reference point *μ*_*k*_ and the uncertainty parameter *σ*_*k*_ do not reflect the true context statistics, evaluation is considered as maladaptive by the RDMA.

**Fig. 2:**
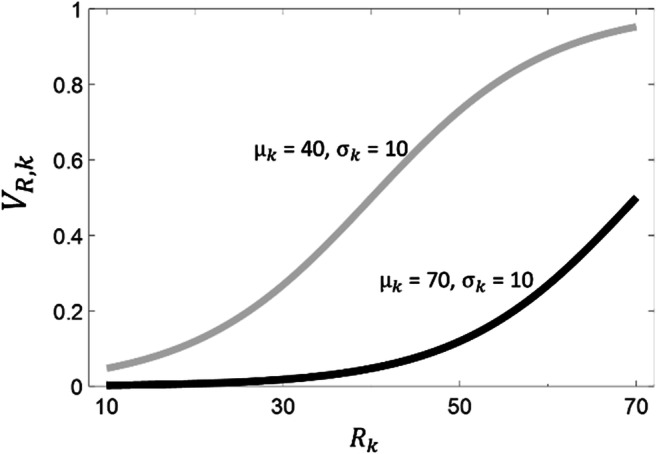
Subjective value as a function of raw value in a context where possible raw amounts are 10, 30, 50, and 70. Value function for different parameter sets is plotted, for a case where parameters reflect the true context statistics (*μ*_*k*_ = 40, *σ*_*k*_ = 10) and a case where the reference point *μ*_*k*_ is high (*μ*_*k*_ = 70, *σ*_*k*_ = 10)

We propose that an excessively high reference point *μ*_*k*_ at play across multiple contexts is at the root of AN. According to the RDMA, what are the implication of an excessively high reference point *μ*_*k*_? Let us consider the example above (describing a context with raw values 10, 30, 50, 70), but now where the reference point *μ*_*k*_ is equal to 70 (Fig. 2), namely substantially higher than the contextual average (which is 40). Comparing the results for *μ*_*k*_ = 40 versus *μ*_*k*_ = 70, two key differences emerge. First, all subjective values are lower when *μ*_*k*_ = 70 (Fig. 2). In other words, a high reference point will lead to considering all possible outcomes as more negative. The second aspect concerns the distance in subjective value among outcomes that are adjacent in the distribution (e.g., 30 minus 10, or 50 minus 30, or 70 minus 50) (Fig. 2). When the reference point *μ*_*k*_ is equal to the contextual average (in our example, μ = 40), this distance is maximal near the average (in our example, it is maximal for 50 minus 30; Fig. 2). Conversely, when the reference point *μ*_*k*_ is high (in our example, μ = 70), this distance is maximal for a region above the average (in our example, it is maximal for 70 minus 50). Moreover, in the lower tail of the contextual distribution, distances among adjacent outcomes are larger when the reference point is close to the contextual average compared to when it is high. In our example, for 50 minus 30 and 30 minus 10, the distance in subjective value is larger when *μ*_*k*_ = 40 compared to when *μ*_*k*_ = 70. In short, when comparing a reference point close to the contextual average versus a high reference point, the distance for adjacent outcomes is larger, except for a region at the high-end of the distribution.

We argue that this scenario can explain general characteristics of AN, including perfectionism, dichotomous thinking, low self-esteem, and low general sense of control (Burns & Fedewa, 2005; Byrne et al., 2008; Egan, Piek, Dyck, & Rees, 2007; Egan, Wade, & Shafran, 2011; Flett & Hewitt, 2002). Perfectionism occurs when all possible outcomes are considered as negative except for only those at the very high-end of the distribution (Egan et al., 2007, 2011; Flett & Hewitt, 2002). Moreover, even these outcomes at the top are usually not experienced with positive feelings, but rather with just a sense of relief. For example, the top mark at school might not be perceived as a great achievement to be celebrated, but simply as the minimum to be expected. This picture of perfectionism fits with the scenario described by the RDMA where a high reference point is implemented. In our example, all outcomes are evaluated as negative (i.e., they have a subjective value less than 0.5) except for the outcome of 70, which is associated with a neutral value (equal to 0.5). This scenario captures the notion that, in perfectionism, expectations (captured by the reference point *μ*_*k*_) are too high, resulting in disappointment (when the outcome is worse than expectations) or, at best, in relief (when the outcome matches expectations).

Dichotomous thinking occurs when possible outcomes are grouped in two opposing categories (Byrne et al., 2008). Moreover, dichotomous thinking is characterised by polarization, namely it maximises the perceived distance among categories of outcomes and minimises the distance within each category (Byrne et al., 2008). This picture of dichotomous thinking also fits with the scenario described by the RDMA where a high reference point is implemented. In our example, comparing the condition where *μ*_*k*_ = 70 versus *μ*_*k*_ = 40, the difference in subjective value is minimised for 10, 30, and 50; in other words, these outcomes are perceived as more similar. Hence, the RDMA predicts that a high reference point will group these outcomes together. At the same time, comparing the condition where *μ*_*k*_ = 70 versus *μ*_*k*_ = 40, the distance between 50 and 70 is enhanced; these outcomes are perceived as more far apart (Fig. 2). Therefore, in line with the notion of dichotomous thinking, the RDMA predicts that a high reference point groups the outcomes of 10, 30, and 50 together, while treating the outcome of 70 as a separate category (note that dichotomous thinking is exacerbated by a low uncertainty parameter *σ*_*k*_, something that also might characterise some AN patients).

Because a higher reference point *μ*_*k*_ implies lower subjective values (see above), low self-esteem (i.e., a low subjective value attributed to the current state (*V*_*pres*, *k*_) averaged across all contexts) also ensues (Brockmeyer et al., 2013). Finally, higher reference point usually entails low general control (Surgenor, Horn, Plumridge, & Hudson, 2002): consider two individuals, with one having higher reference point *μ*_*k*_. Imagine that both individuals predict that an outcome of 50 can be achieved with the correct behaviour and that an outcome of 10 will be achieved without that behaviour. Perceived control will be lower for the individual having higher reference point *μ*_*k*_, because the distance in subjective value between 50 and 10 (corresponding to the level of control; see equation 2) is smaller for this individual.

Why would some people develop a high reference point? Genetic factors might be important, expressed in an inbuilt predisposition for developing higher reference points for evaluation. Social pressure for high standards also might be at play. This might comprise cultural pressure (e.g., exposure to media focusing on highly successful individuals) (Crisp, 1980; Garner & Garfinkel, 1980), group pressure (e.g., experiencing highly competitive schools or sport activities) (Costa-Font & Jofre-Bonet, 2013), and family pressure (e.g., parents teaching their children that the top mark at school is the norm) (Cook & Kearney, 2009, 2014). Moreover, repeated experience of outcomes within a context will normally underpin new learning, leading to a progressive adaptation of the reference point (we do not explore learning here, although this represents an interesting research avenue). However, learning might be impaired for some individuals, resulting in an excessively small learning rate, implying that for these individuals the reference point might fail to adapt and remain abnormally high.

Altogether, within the RDMA, a high reference point at play across contexts elicits perfectionism, dichotomous thinking, low self-esteem, and low general control. RDMA interprets these as all arising from a unique factor (a high reference point), explaining why empirically they are commonly observed together (Burns & Fedewa, 2005; Egan et al., 2007; Kaye et al., 2013). A high reference point is proposed to be at the root of AN and, therefore, of perfectionism, dichotomous thinking, low self-esteem, and low control as observed in the illness. This raises the crucial question: how do these general features (observed also in other mental disorders) result in the specific AN symptoms? The next section examines this question.

## Specific symptoms

Perfectionism, dichotomous thinking, low self-esteem, and low general control are characteristics of AN but also of other disorders, such as obsessive-compulsive disorder (OCD) and depression (Blatt, 1995; Egan et al., 2011; Frost & Steketee, 1997; Orth, Robins, & Roberts, 2008; Seligman, 1974). To understand when, according to the RDMA, such general characteristics result specifically in AN, remember that the RDMA proposes that individuals tend to engage in life contexts where they perceive higher control, and disregard the other contexts (see above). This implies that, when an individual perceives higher control regarding body shape compared to all other life contexts, the individual will focus on shaping the body (Cooper, 2005; Fairburn et al., 1999; Slade, 1982). If the difference in perceived control for body shape compared to all other life contexts is dramatic, this will result in an obsession for body shape (and an exclusive engagement with related activities), which is at the core of AN. More specifically, the RDMA suggests that AN patients have (1) high reference point *μ*_*k*_ for all life contexts including body shape, (2) they attribute low subjective value to the current state *V*_*R*, *k*_ for all contexts including body shape, (3) they attribute low control (defend by equation 2) for all contexts except body shape, and (4) they attribute relatively higher control to the body shape context (i.e., they believe that, with the right commitment, they can achieve a much better outcome (*V*_*act*, *k*_) compared with no commitment (*V*_*Noact*, *k*_)—note that here the difference between *V*_*act*, *k*_ and *V*_*Noact*, *k*_ is critical, and not the individual value of these variables). This argument explains a core symptom in AN, namely the obsession for body shape. We propose that relatively higher control for the body shape is specific to AN: an individual with a similar profile but with relatively higher control for, say, hygiene instead of body shape will not develop AN, but an obsession for hygiene (this reasoning can inspire a future extension of the model to OCD).

However, so far this argument leaves other core symptoms unexplained: why is an extremely low body weight, and not a normal body weight, the target for patients? And why do patients perceive themselves as overweight even when their actual weight is dramatically low (Moelbert et al., 2017)? One possibility compatible with the RDMA is that patients have an extremely low body weight as target already before the illness emerges. This target might be the consequence of repeated exposure to people and media despising body fat and eating, and praising thinness and fasting (Crisp, 1980; Garner & Garfinkel, 1980) (genetic factor might also be at play). However, the RDMA raises another possibility: the patients’ target might not be fixed from the start, but it might decrease as the illness progresses. This decrease might depend on the principle, advocated by reference-dependent cognition models (Kőszegi & Rabin, 2006; Louie et al., 2013, 2015; Rigoli, 2019; Rigoli, Friston, et al., 2016; Stewart, 2009; Stewart et al., 2006), that the reference point changes by tracking changes in the context statistics. Applied to AN, this principle implies the following: at first, patients might have a relatively normal weight as target. When, as described above, an obsession for body shape arises, patients would start dieting to achieve this target. Through selective attention (Blechert, Ansorge, & Tuschen-Caffier, 2010; Jansen, Nederkoorn, & Mulkens, 2005), dieting would expose patients to more and more images of bodies with lower weight. This repeated exposure would lead to a shift in the reference point *μ*_*k*_ towards more and more thinness, changing the patients’ target: now a lower body shape is necessary to achieve the same level of subjective value as before. This explanation entails a vicious cycle, whereby an obsessive diet leads to exposure to skinny body images and in turn to a shift in the reference point, encouraging further dieting. This explains a second core symptom of AN, namely the extremely low body weight combined with a staunch refusal to gain weight. Body misperceptions, a third core symptom (Moelbert et al., 2017), also arise from this argument: according to the notion that perception is not absolute but reference-dependent, a change in the reference point would result in perceiving the own body as overweight despite clear evidence of the contrary. Given that only few people on a diet develop AN, when would dieting lead to developing the specific symptoms of AN? Our reasoning suggests that these symptoms emerge only in some specific circumstances, namely (1) when the diet is obsessive and rigid and (2) when the person dieting is characterised by elevated perfectionism, dichotomous thinking, low self-esteem, low general control, and relatively high control for body shape. However, to some extent, the processes fostering lower target body weight might be promoted by diet also in people who do not develop any pathology.

In short, the RDMA argues that with AN, a low sense of control pervades all life contexts except body shape, resulting in an obsession about the latter. This would trigger a rigid diet associated with exposure to skinny body images, leading to a shift in reference point. Such shift would explain why patients aim at an extremely low body weight and why they perceive themselves as overweight despite evidence of the contrary.

## Discussion

Building on influential accounts of AN, the RDMA offers a computational perspective on this illness. Relying on the idea of reference-dependent evaluation (Kőszegi & Rabin, 2006; Louie et al., 2013, 2015; Rigoli, 2019; Rigoli, Friston, et al., 2016; Stewart, 2009; Stewart et al., 2006), the key proposal is that a high reference point is at the root of general characteristics, such as perfectionism, dichotomous thinking, low self-esteem, and low sense of control. These characteristics would result specifically in AN when the sense of control regarding body shape, compared with other life domains, is relatively high. The model raises the possibility that reference effects also might explain why patients aim at an extremely low weight: exposure to skinny body images (one of the product of obsessive dieting) might change the reference point for the own body; hence leading to extremely low body weigh,t staunch refusal to gain weight, and body misperceptions.

Previous influential theories of AN have emphasised the role of perfectionism and dichotomous thinking in decreasing self-esteem and sense of control (Cooper, 2005; Fairburn et al., 1999; Slade, 1982). These processes would be typical of many girls in their adolescence, when a shift from a dependent to a more independent role is expected, and when the new standards might appear as impossible to achieve (Crisp, 1980). In this paralysing situation, self-starvation would become appealing as a way to establish control and self-esteem at least in one life domain (Cooper, 2005; Fairburn et al., 1999; Slade, 1982). This picture described by previous models is not far from the interpretation offered by the RDMA. The latter contributes to the literature by offering a clear analysis of the key concepts and of their dynamics. First, perfectionism, dichotomous thinking, self-esteem, and control are all framed within a reference-dependent evaluation perspective. A unique factor, namely a high reference point, is proposed to explain all these aspects. This helps understanding why these aspects often appear together empirically (Egan et al., 2007, 2011; Flett & Hewitt, 2002). Second, the RDMA defines these key aspects mathematically rather than verbally (e.g., see equation 2 for the definition of control). Although some simplifications are necessary, mathematical definitions are rigorous, facilitating theoretical debate and formulation of empirical hypotheses (Frank et al., 2016). For example, a novel empirical hypothesis arising from the RDMA is that, as the illness progresses, patients’ weight standards might change because of reference effects. Third, the RDMA offers a mechanistic perspective on how, in specific circumstances (i.e., when the relative control associated with body shape is high), general characteristics (common to several disorders) produce the core symptoms of AN. A formal interpretation of these symptoms emerges: for example, body misperceptions are proposed to arise because of a comparison with an extremely low reference point when making judgements about the own body.

However, despite its similarity to previous models (Cooper, 2005; Fairburn et al., 1999; Slade, 1982), the RDMA is a novel theory, and some of its key tenets remain to be tested. We have already highlighted the possibility that, as AN progresses, patients’ weight standards might change because of reference effects. Two key predictions arise from this. First, weight standards are predicted to decrease as AN progresses and symptoms worsen. Indirect evidence in support of this indicates that, after treatment, patients’ body misperceptions ameliorate, and that this occurs in conjunction with improvements in symptoms (Boehm et al., 2016; Calugi, El Ghoch, Conti, & Dalle Grave, 2018; Roy & Meilleur, 2010). Second, changes in standards are explained as arising from reference effects, a prediction so far unexplored (e.g., this implicates that, for AN patients, repeated exposure to body images with higher or lower weight will affect their standards accordingly). Another key prediction of the RDMA is that, while AN is associated with lower general control (in line with empirical evidence; Kaye et al., 2013), patients perceive relatively higher control for body shape compared to other domains; this key aspect remains to be assessed empirically. This also implicates that events that diminish control for other life domains (e.g., perceived failure at school or sport) will increase the focus upon body shape, whereas events that increase control for other life domains will shift the focus away from body shape. At the same time, events that diminish control for body shape (e.g., perceived failure to lose weight) are predicted to decrease the focus upon body shape (one of the immediate consequences of this could be binge eating), whereas events that increase control for body weight will increase the focus upon body shape. These are all predictions that remain to be examined empirically. Moreover, the RDMA raises specific predictions about the value function (mapping outcomes to subjective values) characterising AN, distinguishing the model from other proposals. For example, the idea of a high reference point (advocated by the RDMA) implies that most outcomes will elicit similar subjective value, but that the very top outcome will prompt a substantial subjective value increase. This contrasts with proposals arguing that a general insensitivity to stimuli (extended also to top outcomes) underlies AN (Davis & Woodside, 2002; Kaye, Frank, Bailer, & Henry, 2005).

The empirical literature highlights two important aspects of AN we have not discussed yet. The first aspect is harm avoidance, reflecting a tendency to adopt avoidant strategies to cope with potential threats (Cassin et al., 2005; Wagner et al., 2006; Kaye et al., 2013). In general, it can be argued that perceiving low control favours avoidance: if no action can manage a potential threat, then avoidance appears as reasonable. The RDMA proposes that, because of a high reference point, AN is associated with low control in all life domains except body shape, hence predicting adoption of avoidant strategies in most life domains (in line with empirical evidence; Cassin et al., 2005; Wagner et al., 2006; Kaye et al., 2013). A second important aspect of AN not discussed yet is alexithymia, combined with impaired interoception (Barca & Pezzulo, 2020; Fassino, Pierò, Gramaglia, & Abbate-Daga, 2004; Kaye et al., 2013; Pollatos et al., 2008; Sexton, Sunday, Hurt, & Halmi, 1998). It has been proposed that alexithymia and impaired interoception emerge because patients do not consider body signals as valuable, hence setting goals that ignore these signals (e.g., pursuing a low body weight even if this entails painful hunger) (Barca & Pezzulo, 2020). With time, disregarding these signals would impair the ability to read them, resulting in alexithymia and impaired interoception (Barca & Pezzulo, 2020). The RDMA does not examine alexithymia and impaired interoception in AN; an interesting avenue is thus to integrate the RDMA with theories examining these aspects.

Although our focus has been on contexts defined by the external environment, our framework can view contexts as arising from a combination of external and internal conditions (Niv, Joel, & Dayan, 2006). For example, the same external environment (e.g., school) can be associated with either being hungry or being satiated, with each motivational state defining a specific context and implying a specific outcome distribution with specific values (e.g., with food being valuable when hungry but not when satiated). Exploring reference effects within contexts defined by internal conditions appears as promising, especially in disorders such as AN where an extremely rigid diet might impact upon parameters governing internally defined contexts.

Research on evaluation highlights two distinct modes of behaviour, goal-directed and habitual (although the debate on how to describe them precisely is ongoing) (Balleine & O'doherty, 2010). During goal-directed behaviour, an individual has a rich representation of the consequences of different courses of actions, while habitual behaviour is driven by automatic stimulus-response associations. It has been suggested that certain forms of mental illness initially emerge from goal-directed processes, but as they chronicize, are then maintained and exacerbated by habitual mechanisms (Everitt & Robbins, 2005; Gillan & Robbins, 2014). Such shift from goal-directed to habitual behaviour has been proposed as critical in the chronicization of AN (O’Hara, Campbell, & Schmidt, 2015). This has implication for the RDMA, where goal-directed and habitual mechanisms are not yet distinguished (more generally, literature on reference effects remains to be integrated with literature distinguishing goal-directed and habitual mechanisms). A promising research avenue is to examine the distinction between goal-directed and habitual mechanisms within the framework offered by the RDMA.

Recent models of AN propose a neuroscientific outlook to understand this disorder. In particular, they highlight the role of impairments in neural reward processes, involving neurotransmitters such as dopamine and serotonin (Kaye et al., 2013; Keating, 2010; O’Hara et al., 2015). This literature emphasises the importance of integrating psychological and neural aspects to fully understand AN. By proposing a formal description of key aspects of AN, the RDMA offers a potential framework for this integration. For example, the RDMA builds on notions such as reward, punishment, and control, that can all be mapped to specific neural mechanisms. An interesting research avenue is to explore the RDMA in the context of neuroscientific literature on AN and to extend the model to the neural level.

## Conclusions

Building upon influential theories of AN, this paper proposes a computational model of the illness, characterising the underlying processes in a formal way. In this way, the model contributes to clarify the meaning of key concepts adopted in the literature and of their relation. Such computational account might help to foster theoretical debate, formulating novel empirical predictions, and to integrate psychological and neuroscientific perspectives on AN. Moreover, this proposal encourages the application of reference-dependent evaluation models to other mental disorders. For instance, depression might result from an excessively high reference point characterising all contexts with no exception (contrary to AN where body shape would represent an exception), producing low self-esteem and low control; whereas OCD might emerge from a very similar profile to AN (in line with the high comorbidity between the two conditions; O'Brien & Vincent, 2003) but from cases where the relative high control is not associated with body shape but with other contexts, such as hygiene, security, or order. Exploring the potential insight on mental illness offered by reference-dependent evaluation models appears as a promising research avenue.
